# TGF-β induces a heart failure phenotype via fibroblasts exosome signaling

**DOI:** 10.1016/j.heliyon.2019.e02633

**Published:** 2019-10-21

**Authors:** Hesham Basma, Adelaide N. Johanson, Kajari Dhar, Daniel Anderson, Fang Qiu, Stephen Rennard, Brian D. Lowes

**Affiliations:** a University of Nebraska Medical Center, USA; b Early Clinical Development, AstraZeneca, Cambridge, UK

**Keywords:** Cell biology, Biochemistry, Regenerative medicine, Oxidative stress, TGF-b, Heart failure, Exosomes

## Abstract

**Purpose:**

The mechanisms for persistent and progressive loss of myocardial function in advanced heart failure (HF) remain incompletely characterized. In the current study, we sought to determine the impact of TGF-β on fibroblasts transcriptional profiles and assess if exosomes from TGF-β treated fibroblasts could induce a heart failure phenotype in co-cultured cardiomyocytes.

**Method:**

Normal heart fibroblasts were treated with TGF-β with a final conc. of 2.5 ng/ml in serum free media. HF fibroblasts were also obtained from patients undergoing implantation of left ventricular assist devices. Exosomes were collected using three-step ultracentrifugation. Cardiomyocytes were co-cultured with exosomes from TGF-β-treated, HF and control fibroblasts. RNA was extracted from the fibroblasts, exosomes, and the cardiomyocytes for a targeted panel of genes using Ion AmpliSeq. Fibroblast function was evaluated by collagen gel contraction.

**Results:**

Fibroblasts treated with TGF-β differentially express 21 of the 140 genes in our targeted panel. These fibroblasts exhibit enhanced collagen gel contraction similar to HF fibroblasts. Fifty of these targeted genes were also differentially expressed in fibroblast exosomes. Pathway analysis of these transcriptional changes suggest hypertrophic signaling to cardiac muscle. Cardiomyocytes, co-cultured with exosomes from TGF- β treated fibroblasts or heart failure patients, differentially expressed 40 genes compared to controls. Cardiomyocytes co-cultured with exosomes of TGF-β treated fibroblasts induced a molecular phenotype similar to cardiomyocytes co-cultured with exosomes from HF fibroblasts. These changes involve contractile proteins, adrenergic receptors, calcium signaling, metabolism and cell renewal.

**Conclusion:**

TGF-β induces broad transcriptional changes in fibroblasts as well as their exosomes. These exosomes induce a heart failure phenotype in cardiomyocytes. Exosome signaling from fibroblasts likely contributes to disease progression in heart failure.

## 1 Introduction

Heart failure is a major cause of morbidity and mortality in the United States. There are approximately 6.0 million Americans living with heart failure representing about 2.8% of the adult US population [1]. There are over 1 million hospitalizations for acutely decompensated heart failure every year, and it remains the largest federal Medicare expenditure. Heart failure can be genetic but is also caused by numerous disease processes, including coronary artery disease, alcohol, viruses and thyroid disease [2]. Heart failure is characterized by transcriptional changes in myocytes that contribute to contractile dysfunction as well as an increase in fibrosis ultimately contributing to diastolic dysfunction and arrhythmia risk [3, 4, 5, 6, 7, 8]. Little is known about the interactions between cardiomyocytes and myocardial fibroblasts in heart failure.

Transforming growth factor beta (TGF-β) is a cytokine that controls a diverse set of cellular processes. TGF-β expression is up-regulated in myocardial infarction, cardiac hypertrophy and in dilated cardiomyopathies [9]. TGF-β transmits its signal through *Smad* dependent and independent pathways [10]. Animal studies suggest that TGF-β induces myosin isoform shifts, one of the key characteristics of heart failure [11]. These molecular changes often persist in heart failure despite standard medical and device therapy with beta-blockers, ACE inhibitors, aldosterone antagonists, and Left Ventricular Assist Devices (LVAD) [7].

Exosomes are 30–150 nM particles released into the extracellular environment through the fusion of multi-vesicular bodies with the plasma membrane by many cell types [12, 13, 14, 15]. This process provides mechanisms for the release of particles that contain cytoplasmic contents including mRNAs and miRNAs. Cells release exosomes under both normal and pathological conditions, and they can be isolated from extracellular fluids, including blood, urine, amniotic fluid, saliva, milk, malignant ascites, synovial fluid and cerebrospinal fluid [16]. In murine models of heart failure, stressed fibroblasts secrete exosomes enriched in miRNAs that cause cardiac hypertrophy [17, 18].

In the current study, we evaluated the direct effects of TGF-β on transcriptional profiles of fibroblasts and their exosomes. We also sought to assess the role played by exosome-mediated signaling from fibroblasts to cardiomyocytes.

## 2 Materials and methods

### 2.1 Cell lines

Primary normal heart cardiac fibroblasts were purchased from Lonza (Walkersville, MD). Cells were maintained in 10%FCS-DMEM containing Penicillin/Strep/Funzigone. Human cardiomyocytes were derived from induced pluripotent stem cells, iCell® cardiomyocytes, (Cellular Dynamics, Madison, WI). iCell® Cardiomyocytes are a mixture of spontaneously electrically active atrial, nodal and ventricular-like myocytes with typical biochemical, electrophysiological, and mechanical characteristics and expected responses upon exposure to exogenous agents. Cells were grown in 48-well plates at 150,000 cells per well according to manufacturer recommendations and maintained in the provided media and starts contract in two days after plating.

### 2.2 Heart failure cells

Failing tissue was obtained from non-ischemic cardiomyopathy patients (n = 3) who met standard criteria for LVAD placement. With IRB approval, the Nebraska Cardiovascular Biobank obtains myocardial tissue from patients receiving LVAD's as destination therapy or bridge to transplant. Fibroblasts were cultured from HF patients' tissue by our established protocol and were harvested under standardized conditions. IRB protocol #133-14-EP was approved by UNMC IRB Committee and informed consent was obtained from all patients.

### 2.3 Ex vivo fibroblasts culture

Tissue from heart failure patients were transferred to 60 mm dish and minced with sterile forceps and scalpel. Cells were cultured in 1.5 ml of 10%FCS-DMEM containing Penicillin/Strep/Funzigone. Media were changed every day until Day 5 then every other day thereafter. At Day 10, fibroblasts were trypsinized and re-plated into a new dish and this was considered passage 1 and maintained after that in 10%FCS-DMEM containing Penicillin/Strep/Funzigone.

### 2.4 TGF-β treatment

TGF-β1 was purchased from R&D Systems (Minneapolis, MN). Primary human normal cardiac fibroblasts were treated with or without TGF-β for 48 h at a final concentration of 2.5 ng/ml in serum free media. Twelve 100 mm dish plates for each experiment (n = 3) were cultured with or without TGF-β. Exosomes from these cells were collected by the three-step centrifugation method. Exosomes were split into two portions. One for isolation of RNA and the other portion was co-cultured with cardiomyocytes. Exosomal RNA was extracted using ExoRNA isolation kit. Targeted panel for heart failure genes were run using Ion AmpliSeq. All experiments were done in triplicates and the mean LogFC of the three runs were compared.

### 2.5 Exosomes isolation and RNA extraction from cells

Primary normal human cardiac fibroblasts were cultured in 10% FCS-DMEM until confluence. For the exosomes experiments, cells were trypsinized and one million cells were plated in 100mm plate (12 plates each for TGF-β and control). After two days, cells were starved for 1hr in serum free media. Fresh serum free media were added, then cells were incubated for two days and media were collected for exosome precipitation. Three centrifugation steps were used to precipitate Exosomes. Briefly, first centrifugation was at 5000 rpm for 60 min followed by two ultra-centrifugation steps at 28000 rpm for 90 min (130,000g). Exosomes from different treatment samples were re-suspended in 500ul of PBS and divided into equal halves, one for targeted panel and the other one for co-culture with cardiomyocytes.

### 2.6 Nanoparticle tracking analysis (NTA)

Extracellular vesicles (EV) concentration and size distribution (size and number of events) were assessed by nanoparticle tracking analysis (NTA) using NanoSight NS300 system (Malvern Instruments, UK. The system is equipped with a 488 nm laser and a syringe pump system, with a pump speed of 20ul/min. Nanoparticles were illuminated by the laser and their movement under Brownian motion was captured. Five individual videos (60 s duration each) for every analyzed sample were recorded and analyzed. The samples were measured with manual shutter and gain adjustments: camera level was set at 14 and an analysis detection threshold of 6 used for every sample to provide the relevant comparisons. Background measurements were performed with filtered PBS, which revealed the absence of any kind of particles. Three samples of each were submitted for analyses. All samples (N = 3 in each group). All samples were diluted with sterile PBS (1:20) in order to reach a particle concentration suitable for analysis with NTA (1.0E+8 to 2.5E+9 particles/ml). The NTA 3.2 software version used to analyze recorded videos. Diluted suspensions were loaded into the sample chamber with 1ml sterile syringes (BD II, New Jersey, USA). Five videos (60 s duration each) of Brownian motion of nanoparticles were recorded and analyzed. The samples were measured with manual shutter and gain adjustments. All measurements were performed at room temperature.

### 2.7 Collagen gel contraction assay

Collagen gels were prepared as described previously [19]. Fibroblasts were trypsinized and mixed with the neutralized collagen solution so that the final cell density in the collagen solution was 3×10^5^ cells/ml. Aliquots (0.5 ml/well) of the mixture of cells in collagen were cast into each well of 24-well tissue culture plates (Falcon), and the mixture was allowed to gel. After gelation was completed, the gels were gently released from the 24-well tissue culture plates and transferred into 60 mm tissue culture dishes (three gels in each dish), which contained 5 ml of freshly prepared serum-free DMEM (SF-DMEM). The gels were then incubated at 37 °C in a 5% CO_2_ atmosphere for 3 days. Gel contraction was quantified using an Optomax V image analyzer (Optomax, Burlington, MA) daily. Data were expressed as a percentage of the initial gel size from three gels per cell line and in three different experiments. Prostaglandin E_2_ (PGE) was purchased from Sigma-Aldrich (St. Louis, MO, USA). PGE was dissolved in 100% ethanol as a stock solution of 10^−3^, and further diluted in medium to 10^−7^ mol/l. Normal heart fibroblasts at 90% confluence were changed to serum free DMEM for 2 h and then treated with PGE for 24 h then with or without TGF-β for 48 h.

### 2.8 Co-culture of exosomes with cardiomyocytes

Exosomes from control normal (n = 3), TGF-β treated (n = 3) and HF (n = 3) cardiac fibroblasts were isolated as mentioned above and were co-cultured with normal cardiomyocytes. Twenty-four hours after co-culture, cardiomyocytes were then trypsinized. RNA was extracted and cardiac target panel were carried out.

### 2.9 Targeted panel

#### 2.9.1 **Constructing RNA library**

Ion AmpliSeq™ RNA Library Kit and Custom Panels were utilized in the construction of RNA library for this investigation, and all experiments were carried out in accordance with the instructions from the manufacturer (Ion Torrent, Life Technologies). Ten nanogram of RNA, isolated from the exosomes, fibroblasts and cardiomyocytes is reverse transcribed to synthesize cDNA using the Ion AmpliSeq™ RNA RT Module and Applied Biosystems thermal cycler. Next target sequences were amplified using Ion AmpliSeq™ RNA Custom Panels and Library Kit (life technologies). Our custom panel was designed to target 140 genes we previously have associated with reverse remodeling in heart failure as well as several candidate genes utilizing Ion AmpliSeq Designer (We excluded five stem cell genes from the results). After amplification of target sequences, primer sequences were partially digested using FuPa reagent from Ion AmpliSeq™ RNA Library Kit. Ion AmpliSeq™ adaptors were then ligated to the targeted DNA and purification of those DNA fragments followed. Target DNA sequences were purified in a two-round purification process with Dynabeads® Magnetic Beads where surplus primer sequences and High-molecular weight DNA are isolated and discarded from the solution. Following purification of the library, the library is amplified using Ion AmpliSeq™ RNA Library Kit and further purified using single-round purification with Dynabeads® Magnetic Beads. After amplification and purification of the library, Qubit® 2.0 fluorometer was used with Qubit® dsDNA HS Assay Kit to quantitatively measure the DNA library.

#### 2.9.2 **Preparation of targeted DNA template and sequencing**

Amplified stock library was diluted for appropriate Ion library preparation. Ion One Touch^TM^2 System was utilized to amplify individual diluted libraries via emulsion PCR on Ion Sphere Particles. The template-positive Ion Sphere Particles (ISPs) were then enriched using Ion One Touch™ Enrichment System. Sequencing was performed for all patients on the Ion Torrent Personal Genome Machine (PGM), utilizing the Ion 316 chip. Coverage analysis as well as mapping the reads and alignment was done using the Ion Torrent Browser Suite™. We have previously validated this targeted gene expression profiles from the PGM sequencer by performing RNAseq with the Illumina Hiseq2500 sequencing system [20, 21].

### 2.10 Functional and pathway analysis in ingenuity pathway analysis (IPA)

To further analyze the biological pathways, differentially expressed genes (p < .05) in exosomes and fibroblasts treated with TGF-β were evaluated by IPA. In addition, differentially expressed genes in cardiomyocytes co-cultured with exosomes of TGF-β treated and HF fibroblasts were plotted into IPA. The analysis illustrates the network for genes that were differentially expressed, their molecular function and their relationship to different diseases including miRNAs, hypertrophy of the heart, apoptosis and necrosis to of cardiac muscles.

### 2.11 Statistical methods

Fibroblasts, exosomes and co-cultured cardiomyocytes RNA samples were sequenced for the HF-selected 140 genes using RNA-seq method. Ion AmpliSeq was performed for HF targeted panel genes. Three samples were treated by exosomes from control fibroblast cells, and 3 samples were treated by exosomes from TGF-β treated fibroblast cells and three. The RNA-sequencing data was filtered to keep the genes with at least nonzero reads for at least 2 samples in one group. After filtering there were 128 genes available for analysis. Significantly up- or down-regulated genes between control and treated groups were identified. The differential expression analysis was conducted using edgeR (Empirical analysis of digital gene expression data in R) package in Bioconductor developed by Robinson et al [22]. The Bejamini-Hochberg method [23] was used to control the false discovery rate (FDR) to be no more than 0.05.

For exosome characterization, unpaired t-test was used to compare the different groups and for gel contraction, paired t-test was used to compare the groups.

## 3 Results

### 3.1 Fibroblasts exosomes characterization

**Fig. 1:**
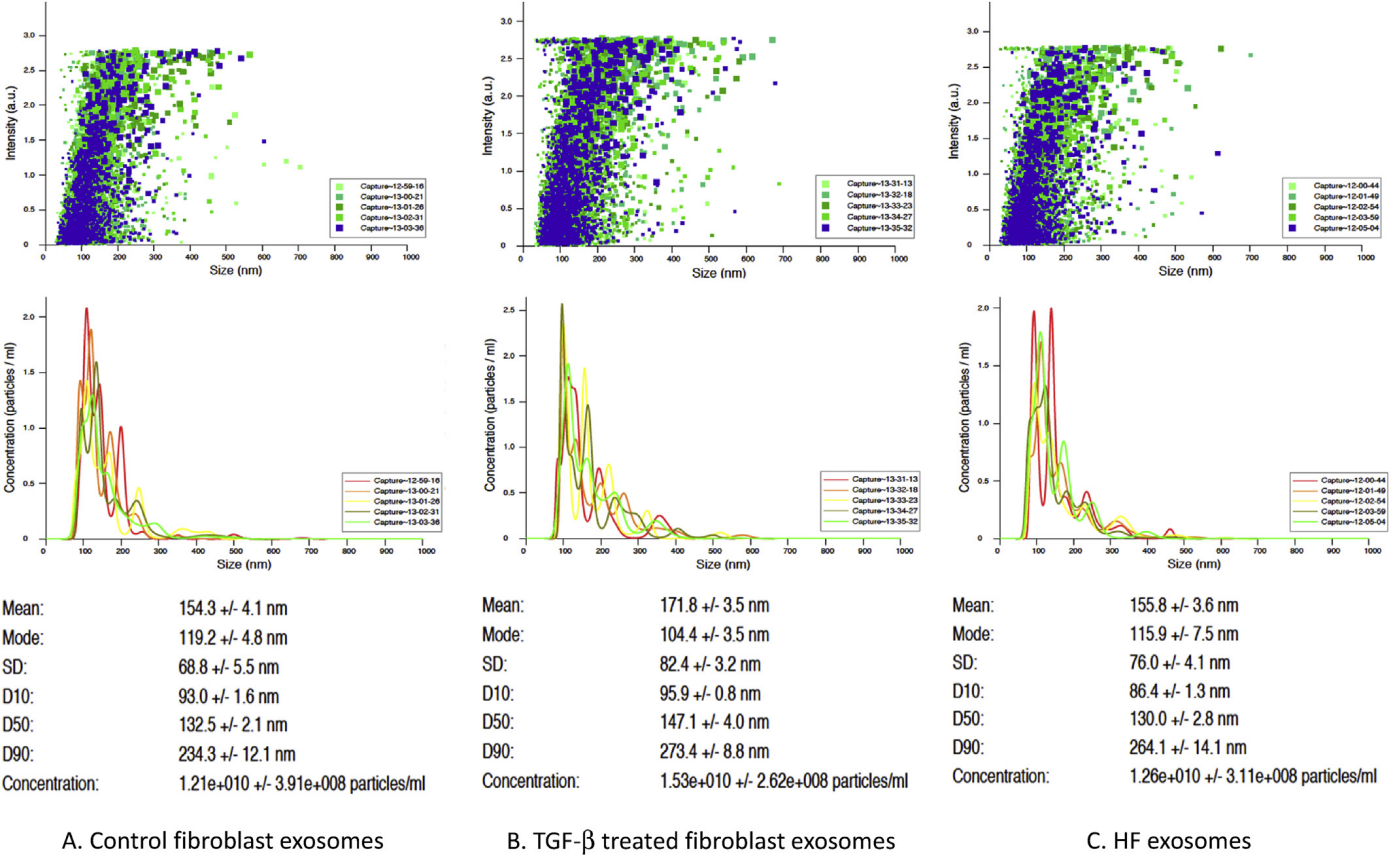
Exosome nanoparticle tracking analysis using NanoSight NS300 system. Five videos (60 s duration each) of Brownian motion of nanoparticles were recorded and analyzed. The samples were measured with manual shutter and gain adjustments (N = 3 in each group). (A). Control fibroblast exosomes. (B). TGF-β fibroblast exosomes. (C) HF fibroblast exosomes.

NanoSight NS300 system was used to characterize exosomes in all samples that were co-cultured with cardiomyocytes (N = 3 in each group). The mean average size of precipitated exosomes in nm were 154.3 +/- 4.1 171.8 +/- 3.5, and 155.8 +/- 3.6 for control, TGF-b treated and HF fibroblasts respectively. Concentration of the exosomes in the solution were 1.21e+010, 1.53e+010, 1.26e+010 for control, TGF-b treated and HF fibroblasts respectively. There was no significant difference in the average size of exosomes and number of events in all the samples (Fig. 1).

### 3.2 TGF-β induced transcriptional changes fibroblasts and exosomes

As shown in Table 1A, 50 genes out of the 140 genes in our targeted panel were differentially expressed in the exosomes treated with TGF-β compared to control exosomes. Furthermore, there were more mRNAs that differentially expressed in exosomes than cells when we compared cells treated with or without TGF-β. In cells, fibroblasts treated with TGF-β differ in only 21 of the target panel genes compared to the control. Nine genes were differentially expressed in both exosomes and cells (Table 1A). Seven genes overlapped in exosomes and cells with similar expression pattern while two were opposite in their expression pattern. There were 41 genes that were differentially expressed in exosomes of fibroblasts treated with TGF-β compared to control but not in fibroblasts and 12 genes that were differentially expressed in fibroblasts but not in their exosomes (Table 1B). These results indicate that exosomes play a pivotal role in cell-cell communication and could serve as a delivery system that delivers not only a single message but rather a full network of communication signals.

**Table 1:** *A*. HF target panel genes differentially expressed in TGF-β treated fibroblasts and their exosomes (N=3 in each group)*.* Genes that were overlapped in both fibroblasts and exosomes and were differentially expressed in TGF-β treated compared to control, up-regulated (bold) and down-regulated (italic) and opposite expression level (bold-italic). *B.* Genes that were differentially expressed in fibroblasts treated with TGF-β compared to control but not in exosomes and that were differentially expressed in exosomes but not in the cells.

A					
No.	Gene ID	Fibroblasts		Exosomes	
		logFC	PValue	logFC	PValue
Genes up-regulated in both					
**1**	**ACTC1**	**3.62579036366492**	**1.15E-06**	**4.34306220386333**	**3.14E-134**
**2**	**ADRB1**	**4.15067746467641**	**0.00045106525616**	**3.58662983153525**	**0.000102019938012**
**3**	**COL1A1**	**2.47423904492034**	**0.000570546020783**	**0.743907933075704**	**0.020136359996399**
**4**	**LDB3**	**2.9632124860499**	**3.23E-05**	**1.38185448721437**	**6.58E-21**
**5**	**TPM1**	**4.10551345735947**	**6.03E-07**	**0.770209933262394**	**0.012942373820731**
Genes down-regulated in both					
*6*	*KCND2*	*-2.74506316216588*	*0.001077509731735*	*-2.12756529627957*	*6.13E-08*
*7*	*MYOZ2*	*-3.35999843083144*	*2.62E-06*	*-2.98880023680181*	*1.51E-65*
Genes with opposite expression level					
***8***	***ACTN2***	***6.06578448673155***	***3.96E-06***	***-0.801875420811882***	***8.58E-05***
***9***	***NPPA***	***4.25915757536294***	***0.005639664808369***	***-4.21802918591052***	***1.53E-06***

B						
Fibroblasts			No.	Exosomes		
Gene ID	logFC	PValue		Gene ID	logFC	PValue
CRY2	1.6726351782109	8.36E-03	1	ABCC9	-1.09572406425757	2.17E-14
JUN	2.78417683699569	4.84E-03	2	ALPL	-1.41077509253921	1.50E-18
KCNIP2	7.03609527206253	5.48E-08	3	CCL2	-1.14833682425684	4.24E-13
MYH6	3.77116708556277	4.07E-03	4	CENPQ	-0.615372155168008	3.12E-04
NFKB2	1.69138565530869	7.75E-03	5	CETP	-0.890917086984853	1.85E-03
PABPC1L	2.64596377213132	1.19E-03	6	CLOCK	-0.35650277263649	1.05E-02
PRPF38B	3.27560062275906	7.55E-05	7	DMD	-0.683525966384984	9.39E-05
RFXAP	3.12742667953124	5.99E-04	8	FPGT	-0.703648294320673	2.19E-05
TAZ	2.45853295533953	1.06E-03	9	HK2	-0.400356916424286	4.78E-03
TNNT2	3.38181281033613	2.80E-05	10	IL1B	-1.32651139448865	1.81E-16
VCL	1.91838305242001	3.01E-03	11	IL8	-1.37742335988644	5.85E-20
VEGFA	2.70240761679433	6.72E-05	12	IRF2	-1.15106670302639	1.90E-14
			13	NFKB1	-0.63849139111603	1.50E-04
			14	NPPB	-0.712579596363141	3.28E-07
			15	PDK4	-2.20859122918407	1.96E-02
			16	PER2	-0.699122984280214	5.00E-05
			17	PER3	-0.964767500776701	4.00E-10
			18	PIK3R1	-0.751483395402562	2.59E-06
			19	PPM1D	-0.632871440676927	4.14E-04
			20	PRKAG2	-0.732622316470305	5.31E-08
			21	RAP2B	-0.572958972427159	1.36E-03
			22	RPS7	-0.387174853351925	1.46E-02
			23	SLC9B2	-0.601755533570746	8.14E-06
			24	SMAD3	-0.827924411520658	4.98E-08
			25	SOAT1	-0.595371811931464	9.46E-06
			26	TBX2	-0.327192758172327	2.16E-02
			27	VTN	-1.59149277204745	6.40E-12
			28	WEE1	-1.26720980348616	3.28E-15
			29	XPNPEP3	-0.484427749366068	3.80E-04
			30	ATRNL1	0.548910996272464	1.09E-04
			31	DSP	0.680847783454209	5.57E-05
			32	EDN1	0.595509958339416	9.18E-05
			33	ERF	0.355272517151082	7.75E-03
			34	GLS	0.503383673453361	2.82E-03
			35	IL6	1.17304888700213	2.12E-10
			36	JUP	1.01001172541033	2.05E-11
			37	MYL2	2.81250831532571	1.31E-04
			38	MYLK	0.455152258426705	7.85E-03
			39	NPAS2	0.553593186395444	1.33E-03
			40	PDIA6	0.432633677021006	6.79E-03
			41	ZNF704	0.661279719253398	2.07E-05

### 3.3 Fibroblasts derived exosomes may contribute to hypertrophy in neighboring cardiomyocytes

**Fig. 2:**
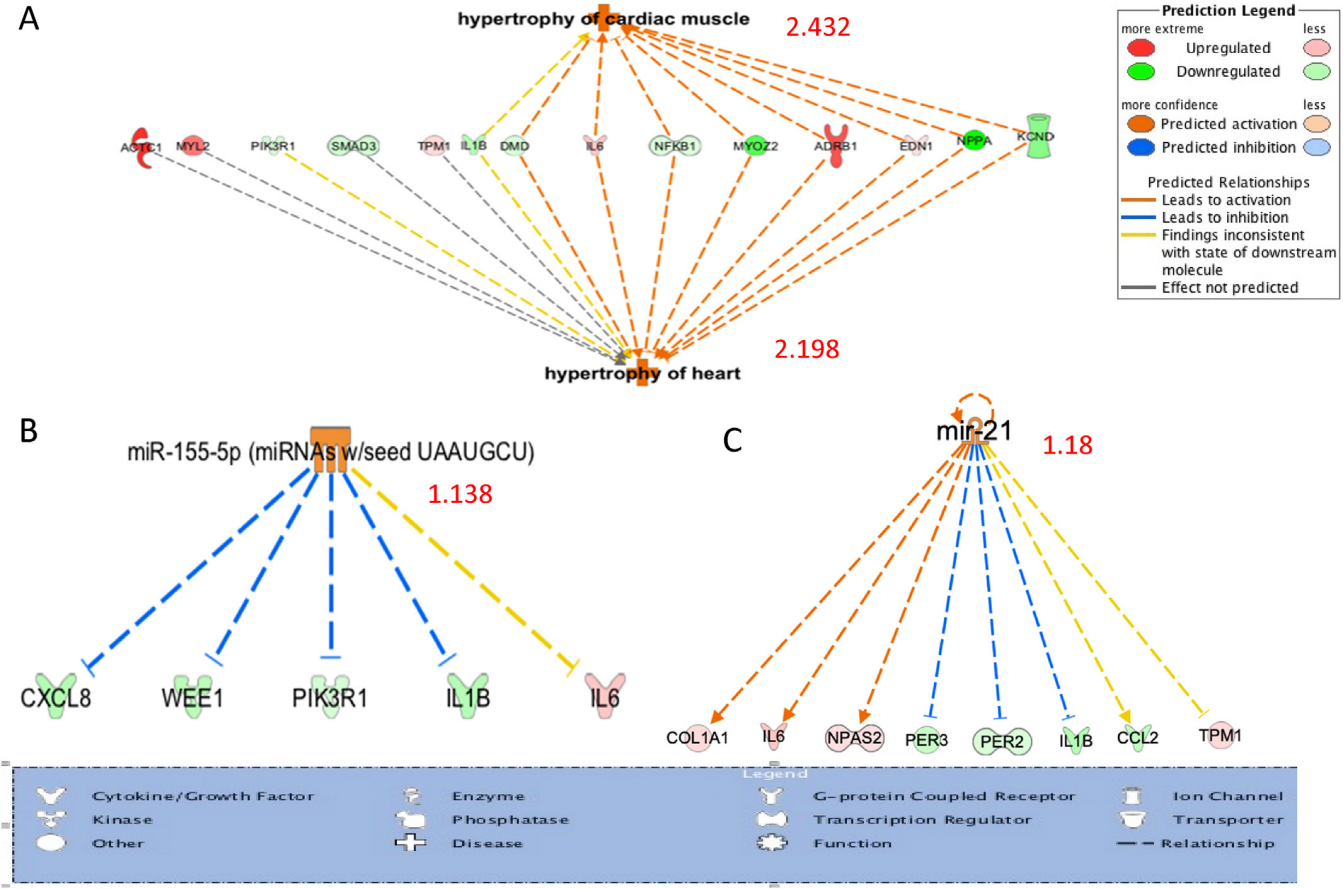
IPA of the differentially expressed genes in fibroblasts exosomes treated with TGF-β compared to control with activation Z-scores. (A). Hypertrophy of the heart (2.198) and cardiac muscle signals (2.432). (B) and (C) mi-RNA 155 (1.138) and 21 (0.88) Z-score.

Ingenuity pathway analysis (IPA) of the differentially expressed genes in TGF-β treated fibroblast exosomes were analyzed. As shown in Fig. 2, hypertrophy of the heart and hypertrophy cardiac muscle were significantly increased in TGF-β treated fibroblast exosomes with activation z-score of 2.198 and 2.432 respectively. Eight molecules out of the 14 molecules were following the prediction while four were not predicted and two were inconsistent with the prediction. Two miRNA upstream analysis showed a potential activation (mir-155 and mir-21) with 1.138 and 1.18 score respectively. The analysis clearly indicates that exosomes of TGF-β treated fibroblasts carry a signal that could increase hypertrophy of the heart. Animal studies suggest that miR21 signals cardiac hypertrophy through exosome signaling [17].

### 3.4 TGF-β treated fibroblast contracted 3-d collagen similar to HF fibroblasts

**Fig. 3:**
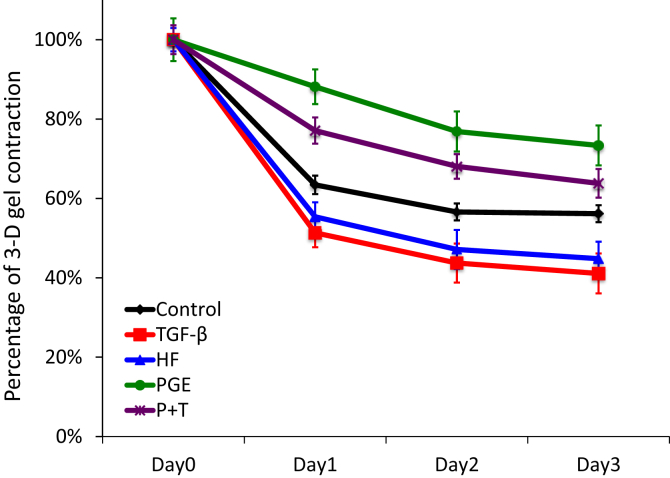
Collagen gel contraction (N = 6 in each group). Control and HF fibroblasts were treated ad cast into collagen gels and maintained in floating culture in serum-free media. The size of gels was measured on day 1, 2 and 3 and shown as percentage of initial area. P + T represents control fibroblasts treated with both PGE and TGF-β.

To assess the functional features of heart fibroblast cells, we evaluated contraction of three-dimensional type I collagen gels mediated by fibroblasts. In addition, we investigated the ability of TGF-β to modulate gel contraction function compared to HF fibroblasts and controls. PGE is a known inhibitor to fibroblast 3-D gel contraction, we utilized it to assess if augmented gel contraction by TGF-β will be inhibited by PGE. TGF-β significantly augmented gel contraction in a similar pattern to HF fibroblasts, 41 versus 45 % on day 3 respectively (Fig. 3). While PGE significantly inhibited collagen gel contraction compared to control, 73% vs 56% on day 3 respectively, PGE inhibited TGF-β gel contraction only partially (64% on day3).

### 3.5 TGF-β and HF fibroblast exosomes co-cultured with cardiomyocytes induce similar gene expression patterns

**Fig. 4:**
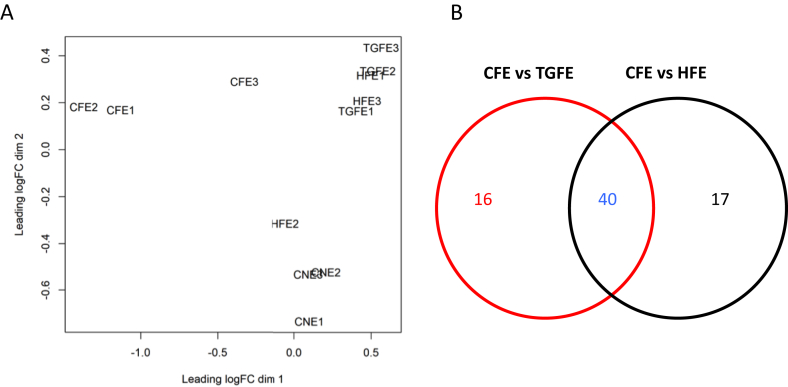
(A). Multidimensional scaling plot of control cardiomyocytes with no exosomes (CNE), co-cultured cardiomyocytes with control fibroblast exosomes (CFE), TGF-β treated fibroblast exosomes (TGFE) and HF fibroblasts exosomes (HFE), (N = 3 in each group). (B). Venn diagram of differentially expressed cells in co-cultured cardiomyocytes with TGF-β treated fibroblast exosomes (TGFE) and HF fibroblasts exosomes (HFE) compared to control fibroblast exosomes (CFE).

Co-cultured cardiomyocytes with exosomes from TGF-β treated and HF fibroblasts were very similar to each other with no significant transcriptional differences compared to each other. Both differed significantly in their target panel genes from their control counter parts co-cultured with control exosomes or control cardiomyocytes with no exosomes. As illustrated in Multidimensional scaling plot of samples and Venn diagram (Fig. 4A and B), co-cultured cardiomyocytes with TGF-β and HF exosomes clustered together and far apart from either cardiomyocytes co-cultured with control exosomes or control cardiomyocytes with no exosomes. To further investigate the similarities and differences between TGF-β and HF co-cultured cardiomyocytes, target panel genes comparison revealed that 40 genes were similar in their expression pattern where 20 genes where upregulated and 20 genes were downregulated in both cell groups compared to controls. Additionally, there were 16 genes uniquely differentially expressed by TGF-β-driven exosomes and 17 genes uniquely driven by HF exosomes compared to controls (Fig. 4B). A list of the 40 genes is illustrated in Table 2A and B).

**Table 2:** A. Target panel genes that were up-regulated in both TGF-β treated fibroblast exosomes and HF fibroblasts exosomes compared to control fibroblast exosomes. B. Target panel genes that were down-regulated in both TGF-β treated fibroblast exosomes and HF fibroblasts exosomes compared to control fibroblast exosomes.

No	Gene ID	Heart Failure		TGF-b	
		Fold Change	PValue	Fold Change	PValue
A					
Genes up-regulated in both					
1	ARNTL2	3.0776227255994	1.24E-03	2.57643382791318	6.92E-03
2	ATP6V1B2	2.66097140334796	0.000248759696666	2.88386223725771	7.7399614742523E-05
3	COQ10B	3.16567497371756	2.39757607160563E-06	4.73441358675273	3.42973662760266E-10
4	COX8A	2.17808350516374	2.99E-04	2.04735940529086	8.56E-04
5	DMD	2.29283640594321	0.000550425891806	2.70241134345075	3.86E-05
6	DSG2	2.06126538687538	4.91E-04	2.14722208321858	2.35E-04
7	ERF	3.21861872425645	0.000137492196678	3.36161669519639	7.99857861885929E-05
8	HK2	3.05542232289123	2.60E-07	1.64699267333126	0.019544372144051
9	LAMP2	3.09172717409777	4.75E-05	2.34231269951093	1.95E-03
10	MYLK	2.22247749568229	0.002926353142711	3.34046303349947	8.60843993773993E-06
11	NFKB1	2.50660138539316	0.00090476049151	2.46910675186973	0.001109482919452
12	PKP2	1.9596630740109	2.01E-03	1.72217376411453	1.24E-02
13	PLN	2.75557965472297	1.01760012939475E-05	2.60985033380605	2.86E-05
14	PPIC	3.20883702251242	2.84E-06	3.13547748108817	4.38E-06
15	PRKAG2	2.19550142029822	0.000148842777967	1.89614670307326	0.001949840630402
16	SPINT2	2.82679478133966	6.25E-05	2.11320940671382	0.003627837620114
17	TGFB1	2.06985142051569	0.004733129127063	2.87298604006755	5.00E-05
18	TMEM43	3.17404640395456	1.30E-06	3.36718776903425	3.85E-07
19	VTN	2.70754137980061	3.66631140296102E-06	1.88288971162895	0.003539509643461
20	WDR44	1.73301233434228	1.98E-02	1.7914760773919	0.01371804289535
B					
Genes down-regulated in both					
1	ACTN2	-2.99443212598882	0.000567940074555	-3.51101080861597	8.88E-05
2	ADRB1	-3.77664463322956	2.19E-04	-4.28285863153771	5.23E-05
3	ATRNL1	-2.99514239403538	4.37992044371054E-05	-2.39160585106481	1.05E-03
4	JUN	-21.7554417199292	1.57E-08	-7.2614334881918	7.99E-05
5	KCNIP2	-21.9344887945276	5.77093281032015E-20	-24.7733635572855	7.22E-21
6	LDB3	-7.2637768024536	6.47E-05	-4.9335762402657	1.01E-03
7	MYBPC3	-6.59976032633372	2.30E-08	-5.70635839730279	1.98E-07
8	MYH6	-2.88016829542143	0.000110446834274	-2.4334301477687	1.07E-03
9	MYL3	-8.58363271526285	9.46E-05	-3.76786292537804	1.16E-02
10	NFKB2	-2.41272209408293	2.81E-05	-2.52868220089862	1.06E-05
11	PABPC1L	-4.13994177252907	2.4336969375533E-09	-8.3914218626246	1.06E-17
12	PCDHB15	-2.38896819622438	7.14E-04	-2.00719514892475	6.14E-03
13	PHF2	-2.16351877267519	0.001086730740856	-2.19729418017628	8.58E-04
14	PLEKHA3	-1.75791582751233	1.83E-02	-1.98838785117807	4.18E-03
15	PRPF38B	-6.46299313570641	1.97E-10	-8.85294784305285	3.01E-13
16	RYR2	-2.53108930782939	1.55227645987285E-05	-2.40435919045305	4.31E-05
17	TAZ	-8.39385573134314	5.77747363180634E-10	-4.7387099778939	2.74E-06
18	TNNI3	-4.28533487976753	3.72E-06	-2.50583339690342	2.79E-03
19	TNNT2	-22.7333085986716	7.90820431782145E-05	-13.1566354564774	7.25E-04
20	VEGFA	-5.52591610376483	1.83E-06	-3.24858662747607	7.32E-04

### 3.6 TGF-β and HF fibroblast exosomes induced a methylation signal in cardiomyocytes

**Fig. 5:**
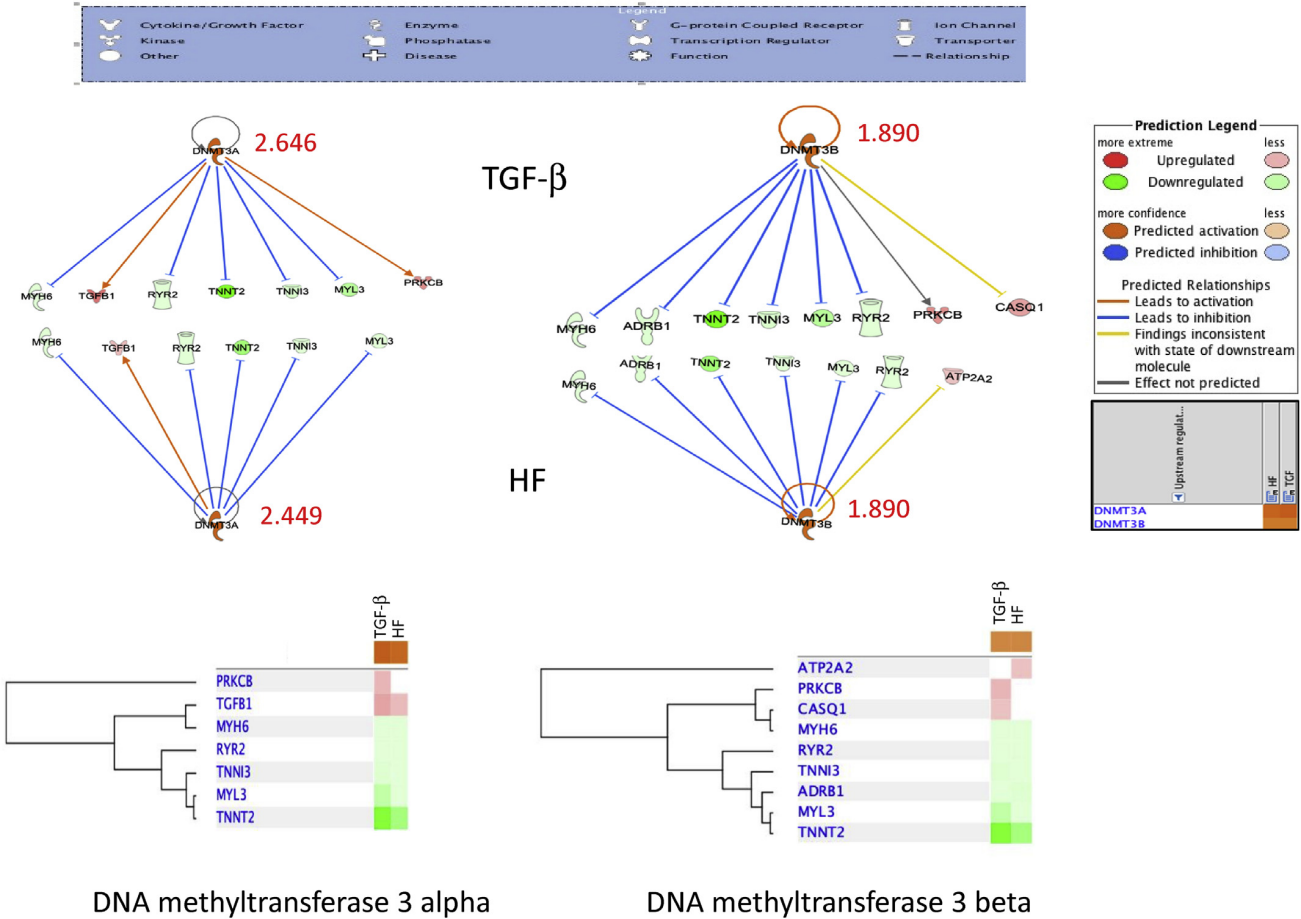
DNA methylation upstream signal in cardiomyocytes co-cultured with of TGF-β treated and HF exosomes. IPA analysis for DNA methyl transferase 3α and 3β and the related genes that were up-regulated and/or downregulated with their z scores in both TGF-β and HF exosomes.

Comparison analysis by IPA for genes in cardiomyocytes co-cultured with TGF and HF fibroblast exosomes revealed that the two highest Z-score were both DNA methyltransferase 3 alpha (DNMT3A) and DNA methyltransferase 3 beta (DNMT3B). In DNMT3α, Z-score was 2.646 and 2.449 in TGF and HF respectively while DNMT3β was 1.890 in both (Fig. 5). This suggests that exosomes in stressed fibroblasts induce a hyper-methylation signal in neighboring cardiomyocytes. Epigenetic changes may play a pivotal role in remodeling changes induced by TGF or HF fibroblast phenotypes.

## 4 Discussion

In this study we determined the impact of TGF-β on transcriptional profiles of fibroblasts and their exosomes. We also evaluated the impact of TGF-β on fibroblast function relative to heart failure and assessed the ability of exosomes from fibroblasts to alter myocyte phenotype. TGF-β is one of the most potent pro-fibrotic cytokines. In the Cardiovascular Health Study, polymorphisms in the TGF-β pathway were strongly associated with Heart Failure preserved Ejection Fraction (HFpEF) suggesting the clinical importance of this pathway in heart failure disease progression [24]. The current study highlights fibroblast to myocyte communication through exosomes and the effect of TGF-β on inducing a heart failure molecular phenotype. We have demonstrated that TGF-β treated fibroblasts and their exosomes differ transcriptionally and functionally from controls. These fibroblasts have enhanced contraction and are producing a hypertrophic signal. A similar observation was previously published from our group in lung fibroblasts significantly stimulates collagen gel contraction in 3D collagen gels in response to TGF-β [25]. In another study by Nagaraju *et al*, HF tissue levels of TGF-β were elevated. Moreover, they found that HF myofibroblasts express high levels of profibrotic cytokines and the TGF-beta1 pathway is activated [26]. In contrast, a recent study by Russo *et al* suggested that TGF-β signaling in activated cardiac fibroblasts were protective against pressure overload-induced remodeling in part through Smad-dependent suppression of matrix-degrading proteases [27].

Fibroblasts and exosome transcriptional changes induced by TGF-β involve upregulation of collagen synthesis (COL1A1), genes that stabilize sarcomere function (LDB3). Interestingly many of the transcriptional changes differed between the fibroblasts and the exosomes. This suggests that the message contained within the exosomes is being selectively packaged or enriched. Pathway analysis of the exosomes suggests that they are signaling myocardial hypertrophy in part through regulation of miR-155 and miR-21. This in concordance with what was previously reported in animal studies indicating that miR-21 is secreted from fibroblasts via exosomes and induces cardiac hypertrophy [17]. Bang *et al* treated fibroblasts with a different profibrotic stimulus, Angiotensin II, that also induced cardiomyocyte hypertrophy via exosome-mediated paracrine messages [17].

Cardiac fibrosis is characteristic for heart failure and prognostic. TGF-β treated cardiac fibroblasts contract three-dimensional collagen gel in a similar fashion as HF fibroblasts. Lijnen et.al reported that TGF-β mediated gel contraction through α-SMA in rat cardiac fibroblasts is dose dependent [28]. Prostaglandin E normally inhibits fibroblast gel contraction and our results suggest it is effective for partially blocking TGF-β activation of fibrosis. It is unknown if PG-E would be effective in the treatment of heart disease. A large cohort study utilizing the U.S Veterans Affairs database suggests that misoprostol reduced the risk of NSAID induced cardiovascular, cerebrovascular and reno-vascular adverse events [29]. Misoprostol is a synthetic PG-E analogue normally used to treat ulcers, initiate labor and to control post-partum bleeding.

Cardiomyocytes on the other hand respond to exosome signaling by developing a transcriptional changes characteristic of heart failure. At a molecular level, heart failure is characterized by broad changes in myocardial gene expression as well epigenetic factors that regulate transcriptional changes. Previous studies have suggested that these changes involve adrenergic signaling, calcium handling, metabolism, inflammation, and contractile proteins [3, 20]. Many of these changes are regulated by miRNAs, small non-coding RNA molecules that can silence mRNA. This study shows that fibroblasts exosomes obtained from heart failure patients can induce a molecular phenotype of heart failure in normal cardiomyocytes. These changes include down regulation of α-MHC, beta-1 adrenergic receptors, and the ryanodine receptor. Exosomes from TGF- β treated fibroblasts produce a similar effect on multiple genes. MYH6 and ADRB1 down-regulation are key components of contractile dysfunction in heart failure [4, 5]. Altered MYH6 expression is an important pathway in the molecular mechanism of heart failure and myocardial recovery. Pathway analysis of altered genes suggest DNA methylation may contribute to these changes. Previous heart failure studies have suggested the importance of DNA methylation transcriptional changes that can be both adaptive as well as maladaptive [30, 31, 32].

This study has several limitations. We utilized a targeted gene expression panel to evaluate transcriptional changes in order to limit false discovery. TGF- β likely induces numerous additional transcriptional changes that were not measured and could be contributing to changes in fibroblast and myocyte function. We also did not measure mi-RNAs directly. Further studies are necessary to better define the role of exosomes on disease progression in heart failure that will require more global analysis of gene expression, as well as the epigenetic factors that regulate them including, miRNAs, long noncoding RNAs and DNA methylation.

In conclusion, TGF-β induces transcriptional changes in fibroblasts as well as their exosomes. These changes functionally trigger increased fibroblast contraction and signal a hypertrophic response to cardiomyocytes via exosomes. The cardiomyocyte expression profile induced by these exosomes is like the molecular phenotype of heart failure. Fibroblast exosomes obtained from patients with advanced heart failure illicit a similar response. Exosome signaling from fibroblasts likely contributes to disease progression in heart failure. There is a need for additional heart failure therapies and fibroblasts should be considered as a target for new approaches. This in vitro model of interactions between human fibroblasts and cardiomyocytes could be utilized to evaluate therapies and study reprogramming of cells in heart failure. Additionally, exosomes carry an important message and should be considered as biomarkers.

## Declarations

### Author contribution statement

H. Basma: Conceived and designed the experiments; Performed the experiments; Analyzed and interpreted the data; Contributed reagents, materials, analysis tools or data; Wrote the paper.

A. Johanson: Performed the experiments.

K. Dhar: Performed the experiments; Analyzed and interpreted the data.

D. Anderson: Contributed reagents, materials, analysis tools or data.

F. Qiu: Analyzed and interpreted the data.

S. Rennard: Analyzed and interpreted the data; Wrote the paper.

B. Lowes: Conceived and designed the experiments; Analyzed and interpreted the data; Wrote the paper.

### Funding statement

This work was supported by the Angle Endowment and the University of Nebraska Medical Center Division of Cardiology.

### Competing interest statement

The authors declare no conflict of interest.

### Additional information

No additional information is available for this paper.
